# Prognostic nutritional index, Naples prognostic score and Osaka prognostic score as predictors of acute respiratory distress syndrome in non-diabetes mellitus patients with sepsis: a loss of predictive efficacy of NPS and OPS in diabetes mellitus cohorts

**DOI:** 10.3389/fendo.2026.1760773

**Published:** 2026-04-22

**Authors:** Jie Wu, Wenfeng Qiu, Xiuwen Lin, Jinrun Wu, Renyuan Li

**Affiliations:** Intensive Care Unit, Meizhou People’s Hospital, Meizhou Academy of Medical Science, Meizhou, China

**Keywords:** acute respiratory distress syndrome, Naples prognostic score, Osaka prognostic score, prognostic nutritional index, sepsis

## Abstract

**Background:**

Acute respiratory distress syndrome (ARDS) induced by sepsis is associated with uncontrolled immunoinflammatory responses and nutritional status. The purpose of this study was to explore the value of several prognostic scoring indices (Prognostic nutritional index (PNI), Naples prognostic score (NPS), and Osaka prognostic score (OPS)) in predicting ARDS risk among sepsis patients.

**Methods:**

2089 sepsis patients were retrospectively enrolled, and divided into diabetes mellitus and non-diabetes mellitus groups based on the presence of comorbid diabetes mellitus. Baseline clinical data and laboratory test indicators at admission were collected, and the PNI, NPS, and OPS were calculated. The differences in PNI, NPS, and OPS between ARDS and non-ARDS patients were compared. Logistic regression analysis was performed to explore the associations of PNI, NPS, and OPS with ARDS in septic patients.

**Results:**

1772 patients did not develop ARDS, whereas 317 cases with ARDS. PNI level in ARDS patients was lower than that in patients without ARDS (*p* < 0.001). ARDS patients had notably higher proportions of NPS scores of 3 or 4 points and OPS scores of 2 or 3 points compared with non-ARDS patients (*p* < 0.001). The proportion of diabetes mellitus was significantly lower in the ARDS group than non-ARDS group (*p* = 0.030). In non-diabetes mellitus patients, logistic regression analysis revealed that low PNI (odds ratio [OR]: 3.764, 95% confidence interval [CI]: 2.549-5.557, *p* < 0.001), NPS score 3-4 (OR: 2.537, 95% CI: 1.302-4.944, *p* = 0.006), and OPS score 2-3 (OR: 3.189, 95% CI: 1.326-7.670, *p* = 0.010) were independently associated with ARDS. In patients with diabetes mellitus, low PNI (OR: 2.037, 95% CI: 1.256-3.306, *p* = 0.004) was independently associated with ARDS, however, neither NPS nor OPS yielded statistically significant results.

**Conclusions:**

PNI, NPS, and OPS were predictive indicators for ARDS risk in sepsis patients without diabetes mellitus; however, NPS and OPS lack corresponding predictive value in diabetes mellitus cohorts.

## 1 Introduction

Sepsis is a life-threatening organ dysfunction disease caused by dysregulation of the host response triggered by infection, and it is a major challenge in the field of critical care medicine (1). Sepsis has a high global prevalence. According to statistics, there are approximately 50 million new cases of sepsis worldwide each year, and more than 20% of these patients die from sepsis and its complications (2, 3). It is worth noting that the elderly population and patients with chronic underlying diseases (such as diabetes mellitus, chronic obstructive pulmonary disease, and malignant tumors.) are at a higher risk of developing sepsis (4).

Acute respiratory distress syndrome (ARDS) is characterized by damage to the alveolar capillary membrane as its core pathological feature (5, 6). ARDS is an acute respiratory failure caused by internal or external factors in the lungs, characterized by persistent hypoxemia and bilateral lung infiltrates, and cannot be fully explained by heart failure or excessive volume load (7, 8). The incidence rate of ARDS varies across different etiologies, and sepsis is the leading cause of ARDS (9, 10). The incidence rate of ARDS in patients with sepsis is approximately 30%-50%, significantly higher than that in cases of trauma, pneumonia, and pancreatitis (11, 12). Accurately identifying high-risk individuals for ARDS and implementing early intervention is crucial for improving prognosis.

In clinical practice, prognostic scoring systems are important tools for risk stratification and prognosis assessment (13). Among them, indicators such as the Prognostic nutritional index (PNI), Naples prognostic score (NPS) and Osaka prognostic score (OPS) have the advantages of being easy to obtain and having high assessment efficiency. The PNI, NPS, and OPS are all comprehensive prognostic assessment tools based on the body’s nutritional status and inflammatory response (14–16). Among them, PNI takes serum albumin level combined with peripheral blood lymphocyte count as core indicators (17). By quantifying the body’s nutritional reserve and cellular immune function, it has shown stable value in the prognostic evaluation of various diseases including tumors, infections, and critical illnesses, and can effectively predict postoperative recovery, infectious complications, and long−term survival in patients (18, 19). NPS and OPS further enhance the coupling effect of inflammatory and nutritional indicators. NPS quantifies the correlation between systemic inflammatory response and nutritional consumption using the neutrophil-to-lymphocyte ratio (NLR), lymphocyte-to-monocyte ratio (LMR), and albumin (20). OPS comprehensively analyzes C-reactive protein (CRP), albumin, and lymphocyte levels to reflect the degree of inflammation-nutrition imbalance (21). The value of NPS and OPS in the prognostic assessment of diseases such as malignant tumors and cardio-cerebrovascular diseases has been verified (16, 20–22). The PNI, NPS, and OPS were initially developed as prognostic tools for oncological patients, primarily to stratify risk and guide management decisions in surgical or advanced cancer settings. However, given the shared systemic inflammatory and nutritional perturbations observed in sepsis and ARDS, these scores have gained attention as potential predictive biomarkers in non-oncological critical care populations.

Diabetes mellitus, as a common chronic metabolic disorder, significantly alters the pathophysiological process of sepsis through various mechanisms such as modifying the inflammatory response pattern, damaging immune function, and affecting vascular endothelial integrity (23). The results of existing studies showed that the pathogenesis, clinical phenotype, and prognosis of sepsis in diabetes mellitus patients differ significantly from those in non-diabetes mellitus patients (24, 25). However, there is currently insufficient research evidence regarding the predictive value of PNI, NPS and OPS for ARDS in patients with diabetes complicated with sepsis. Moreover, preliminary explorations have shown that these factors may lose their predictive efficacy in this specific population.

Given the clinical uniqueness of patients with diabetes mellitus complicated by sepsis and the urgent need for the prevention and treatment of ARDS, it is of great significance to clarify the applicability deficiencies of the existing prognostic scoring systems in this population. Therefore, investigating the differences in the predictive value of PNI, NPS, and OPS for the risk of ARDS between septic patients with and without diabetes mellitus, and optimizing the risk prediction strategy for ARDS in septic patients with different underlying diseases, have become urgent issues to be addressed in current clinical research. Meanwhile, it also provides the core background and research entry point for the present study. The purpose of this study was to explore the value of PNI, NPS, and OPS in predicting ARDS risk among sepsis patients.

## 2 Materials and methods

### 2.1 Study cohort

This study adopted a retrospective cohort study design, with the patients with sepsis admitted to intensive care unit (ICU) of Meizhou People’s Hospital from January 2019 to August 2025 as the research subjects.

The inclusion criteria of patients were as follows (1): aged ≥ 18 years (2); meeting the diagnostic criteria for sepsis, i.e., confirmed or highly suspected infection accompanied by organ dysfunction with a Sequential Organ Failure Assessment (SOFA) score ≥2 points (3); length of hospital stay ≥24 hours; and (4) complete clinical data available, including demographic characteristics, underlying diseases, laboratory test indicators, infection-related parameters and prognostic information, which allow the calculation of the PNI, NPS and OPS. Exclusion criteria of patients were as follows (1): confirmed ARDS on admission, or presence of severe underlying lung diseases such as acute exacerbation of chronic obstructive pulmonary disease (AECOPD) and pulmonary fibrosis (2); complicated with end-stage diseases including advanced malignant tumors, end-stage renal disease, and decompensated liver cirrhosis (3); pregnant or lactating women (4); missing key indicators in clinical data or obvious logical errors in data; and (5) patients who died or discharged voluntarily within 24 hours of hospitalization. This study was approved by the Medical Ethics Committee of the Meizhou People’s Hospital.

### 2.2 Grouping

Patients were divided into the ARDS group and non-ARDS group based on whether ARDS developed within 28 days after admission. ARDS is diagnosed according to the Berlin Definition of 2012 and must meet the following criteria: (1) acute onset: New or worsening respiratory symptoms within one week; (2) chest imaging findings: bilateral pulmonary infiltrates on chest X-ray or computed tomography (CT), which cannot be fully explained by pleural effusion, atelectasis, or other alternative causes; (3) hypoxemia severity classification: the ratio of arterial partial pressure of oxygen to fraction of inspired oxygen (PaO_2_/FiO_2_), combined with the level of positive end-expiratory pressure (PEEP), is used to categorize ARDS into three grades: mild (200 mmHg < PaO_2_/FiO_2_ ≤ 300 mmHg with PEEP/CPAP ≥ 5 cmH_2_O), moderate (100 mmHg < PaO_2_/FiO_2_ ≤ 200 mmHg with PEEP ≥ 5 cmH_2_O), and severe (PaO_2_/FiO_2_ ≤ 100 mmHg with PEEP ≥ 5 cmH_2_O); (4) Etiology of pulmonary edema: The condition cannot be fully attributed to heart failure or fluid overload.

### 2.3 Data collection and processing

Clinical data of the study subjects were extracted from the electronic medical record system of Meizhou People’s Hospital. The clinical data included age, gender, body mass index (BMI), history of smoking, history of alcohol drinking, hypertension, diabetes mellitus, invasive mechanical ventilation, and laboratory test results (serum albumin, peripheral neutrophil count, lymphocyte count, monocyte count, total cholesterol, and C reactive protein (CRP)). In line with Chinese criteria, BMI is divided into three grades: <18.5 kg/m^2^ (underweight), 18.5-23.9 kg/m^2^ (normal weight), and ≥24.0 kg/m^2^ (overweight) (26, 27). Blood test data were collected during the first hospital examination.

The PNI score is calculated based on the serum albumin level (g/L) and the peripheral blood lymphocyte count (×10^9^/L). The calculation formula is as follows: PNI=serum albumin (g/L) + 5 × lymphocyte count (×10^9^/L).

The NPS was defined as: ①serum albumin (≥40 g/L: 0 point, <40 g/L: 1 point); ②total cholesterol (>180mg/dL: 0 point, ≤180 mg/dL: 1 point); ③neutrophil to lymphocyte ratio (NLR) (≤2.96: 0 point, >2.96: 1 point); ④lymphocyte to monocyte ratio (LMR) (>4.44: point, ≤4.44: 1 point) (28). NPS score is the sum of the scores of albumin, total cholesterol, NLR, and LMR. NPS were categorized into 3 groups: group 0 (score of 0), group 1 (score 1 or 2), and group 2 (score of 3 or 4) (29).

The OPS was computed using three parameters: CRP (0 point for ≤ 10.0 mg/L; 1 point for > 10.0 mg/L), albumin (0 point for ≥35 g/L; 1 point for <35 g/L) and lymphocyte count (0 point for ≥1.6×10^9^/L; 1 point for <1.6×10^9^/L). The OPS represents the cumulative score derived from these three indicators (21).

### 2.4 Statistical analysis

Statistical analysis was performed using SPSS 26.0 software, and GraphPad Prism 9.0 was used for plotting statistical graphs. For continuous variables, if they conformed to a normal distribution with homogeneous variance, data were presented as mean ± standard deviation (SD), and comparisons between groups were conducted using the independent samples t-test. If the data were not normally distributed, they were expressed as median (interquartile range) [M (Q_1_, Q_3_)], and the Mann-Whitney U test was applied for intergroup comparisons. Categorical variables were presented as case number (percentage) [n (%)], and comparisons between groups were performed using the chi-square test (χ^2^ test) or Fisher’s exact test. The receiver operating characteristic (ROC) curve was used to evaluate the predictive value of PNI for ARDS development in sepsis patients, and the area under the curve (AUC), optimal cut-off value, sensitivity, and specificity were calculated. Logistic regression analysis was adopted to identify independent risk factors for ARDS in sepsis patients. *p* < 0.05 was considered statistically significant.

## 3 Results

### 3.1 Comparison of the clinical features between patients with and without ARDS in patients with sepsis

Of the total 2089 patients, 1583 (75.8%) were male and 506 (24.2%) were female. Regarding age distribution, 671 (32.1%) patients were <60 years, while 1418 (67.9%) were ≥60 years old. In terms of BMI, nearly half of the patients (1052/2089, 50.4%) had a normal weight, and 281 (13.5%) were underweight. The prevalence rates of smoking history, alcohol drinking history, hypertension, and diabetes mellitus were 20.9% (436/2089), 9.2% (192/2089), 42.5% (888/2089), and 41.6% (868/2089), respectively. For the NPS, the number of patients with scores of 0, 1, 2, 3, and 4 was 10 (0.5%), 71 (3.4%), 267 (12.8%), 577 (27.6%), and 1164 (55.7%), respectively. Correspondingly, the distribution of OPS was as follows: 32 (1.5%) patients with a score of 0, 249 (11.9%) with 1, 667 (31.9%) with 2, and 1141 (54.6%) with 3 (**Table 1**).

**Table 1:** Comparison of the clinical features between patients with and without ARDS in patients with sepsis.

Clinical characteristics	Total (n=2089)	Without ARDS (n=1771)	With ARDS (n=318)	*p* (χ^2^/Z)
Gender				
Male, n(%)	1583 (75.8%)	1334 (75.3%)	249 (78.3%)	0.257 (χ^2^ = 1.302)
Female, n(%)	506 (24.2%)	437 (24.7%)	69 (21.7%)	
Age (years old)				
<60, n(%)	671 (32.1%)	568 (32.1%)	103 (32.4%)	0.948 (χ^2^ = 0.012)
≥60, n(%)	1418 (67.9%)	1203 (67.9%)	215 (67.6%)	
BMI (kg/m^2^)				
Underweight, n (%)	281 (13.5%)	245 (13.8%)	36 (11.3%)	0.847 (χ^2^ = 0.353)
Normal weight, n (%)	1052 (50.4%)	923 (52.1%)	129 (40.6%)	
Overweight, n (%)	667 (31.9%)	590 (33.3%)	77 (24.2%)	
Unknown, n (%)	89 (4.3%)			
History of smoking				
No, n(%)	1653 (79.1%)	1405 (79.3%)	248 (78.0%)	0.600 (χ^2^ = 0.296)
Yes, n(%)	436 (20.9%)	366 (20.7%)	70 (22.0%)	
History of alcohol drinking				
No, n(%)	1897 (90.8%)	1600 (90.3%)	297 (93.4%)	0.091 (χ^2^ = 3.008)
Yes, n(%)	192 (9.2%)	171 (9.7%)	21 (6.6%)	
Hypertension				
No, n(%)	1201 (57.5%)	1009 (57.0%)	192 (60.4%)	0.268 (χ^2^ = 1.278)
Yes, n(%)	888 (42.5%)	762 (43.0%)	126 (39.6%)	
Diabetes mellitus				
No, n(%)	1221 (58.4%)	1017 (57.4%)	204 (64.2%)	0.026 (χ^2^ = 5.021)
Yes, n(%)	868 (41.6%)	754 (42.6%)	114 (35.8%)	
Invasive mechanical ventilation				
No, n (%)	1199 (57.4%)	1185 (66.9%)	14 (4.4%)	<0.001 (χ^2^ = 430.782)
Yes, n (%)	890 (42.6%)	586 (33.1%)	304 (95.6%)	
SOFA score	5.0 (3.0, 8.0)	4.0 (3.0, 7.0)	7.0 (5.0, 11.0)	<0.001 (Z=-6.257)
PNI, median (IQR)	36.80 (31.55, 43.10)	37.65 (32.50, 43.80)	32.28 (28.08, 36.95)	<0.001 (Z=-10.827)
NPS				
0, n(%)	10 (0.5%)	9 (0.5%)	1 (0.3%)	<0.001 (χ^2^ = 48.767)
1, n(%)	71 (3.4%)	68 (3.8%)	3 (0.9%)	
2, n(%)	267 (12.8%)	250 (14.1%)	17 (5.3%)	
3, n(%)	577 (27.6%)	511 (28.9%)	66 (20.8%)	
4, n(%)	1164 (55.7%)	933 (52.7%)	231 (72.6%)	
OPS				
0, n(%)	32 (1.5%)	29 (1.6%)	3 (0.9%)	<0.001 (χ^2^ = 74.115)
1, n(%)	249 (11.9%)	241 (13.6%)	8 (2.5%)	
2, n(%)	667 (31.9%)	601 (33.9%)	66 (20.8%)	
3, n(%)	1141 (54.6%)	900 (50.8%)	241 (75.8%)	

ARDS, acute respiratory distress syndrome; BMI, body mass index; SOFA, Sequential Organ Failure Assessment score; PNI, prognostic nutritional index; NPS, naples prognostic score; OPS, Osaka prognostic score; IQR, interquartile range.

In the present study, 1772 (84.8%) patients did not develop ARDS, whereas 317 (15.2%) cases were diagnosed with ARDS. The proportion of diabetes mellitus was significantly lower in the ARDS group than in the non-ARDS group (36.0% vs. 42.6%, *p* = 0.030). In contrast, the rate of invasive mechanical ventilation was remarkably higher among ARDS patients compared with non-ARDS counterparts (95.6% vs. 33.1%, *p* < 0.001). Regarding prognostic scoring indices, the PNI level in ARDS patients was substantially lower than that in patients without ARDS (32.25 (28.05, 36.88) vs. 37.68 (32.51, 43.80), *p* < 0.001). Significant differences were observed in the distribution of both the NPS and OPS between the two groups (both *p* < 0.001). Specifically, ARDS patients had notably higher proportions of NPS scores of 3 or 4 points and OPS scores of 2 or 3 points when compared with non-ARDS patients. No statistically significant differences were detected between the ARDS and non-ARDS groups in terms of gender, age, BMI distribution, as well as the proportions of smoking history, alcohol drinking history, and hypertension (**Table 1**).

### 3.2 Comparison of the clinical features between patients with and without ARDS in sepsis patients without diabetes mellitus

In patients without diabetes mellitus (n=1221), there were 1018 patients without ARDS and 203 patients with ARDS. The proportion of patients who have received invasive mechanical ventilation in patients with ARDS was higher than those without ARDS (97.5% vs. 33.9%, *p* < 0.001). The PNI level in patients with ARDS was significantly lower than that in non-ARDS patients (31.95 (27.80, 36.15) vs. 39.73 (33.94, 45.61), *p* < 0.001). The proportions of ARDS patients with an NPS of 3 or 4 points (*p* < 0.001) and an OPS of 2 or 3 points (*p* < 0.001) were significantly higher than those in non-ARDS patients (**Table 2**).

**Table 2:** Comparison of the clinical features between patients with and without ARDS in patients without diabetes mellitus.

Clinical characteristics	Total (n=1221)	Without ARDS (n=1017)	With ARDS (n=204)	*p* (χ^2^/Z)
Gender				
Male, n(%)	938 (76.8%)	776 (76.3%)	162 (794%)	0.364 (χ^2^ = 0.922)
Female, n(%)	283 (23.2%)	241 (23.7%)	42 (20.6%)	
Age (years old)				
<60, n(%)	406 (33.3%)	340 (33.4%)	66 (32.4%)	0.807 (χ^2^ = 0.089)
≥60, n(%)	815 (66.7%)	677 (66.6%)	138 (67.6%)	
BMI (kg/m^2^)				
Underweight, n (%)	202 (16.5%)	169 (16.6%)	33 (16.2%)	0.379 (χ^2^ = 1.953)
Normal weight, n (%)	616 (50.5%)	538 (52.9%)	78 (38.2%)	
Overweight, n (%)	355 (29.1%)	310 (30.5%)	45 (22.1%)	
History of smoking				
No, n(%)	945 (77.4%)	791 (77.8%)	154 (75.5%)	0.521 (χ^2^ = 0.508)
Yes, n(%)	276 (22.6%)	226 (22.2%)	50 (24.5%)	
History of alcohol drinking				
No, n(%)	1112 (91.1%)	923 (90.8%)	189 (92.6%)	0.423 (χ^2^ = 0.746)
Yes, n(%)	109 (8.9%)	94 (9.2%)	15 (7.4%)	
Hypertension				
No, n(%)	836 (68.5%)	699 (68.7%)	137 (67.2%)	0.680 (χ^2^ = 0.195)
Yes, n(%)	385 (31.5%)	318 (31.3%)	67 (32.8%)	
Invasive mechanical ventilation				
No, n (%)	678 (55.5%)	673 (66.2%)	5 (2.5%)	<0.001 (χ^2^ = 279.411)
Yes, n (%)	543 (44.5%)	344 (33.8%)	199 (97.5%)	
SOFA score	5.0 (3.0, 8.0)	4.0 (2.0, 8.0)	7.0 (5.0, 10.5)	<0.001 (Z=-4.597)
PNI, median (IQR)	38.40 (32.40, 44.80)	39.75 (33.93, 45.63)	32.03 (27.88, 36.19)	<0.001 (Z=-10.893)
NPS				
0, n(%)	6 (0.5%)	5 (0.5%)	1 (0.5%)	<0.001 (χ^2^ = 67.912)
1, n(%)	56 (4.6%)	54 (5.3%)	2 (1.0%)	
2, n(%)	198 (16.2%)	189 (18.6%)	9 (4.4%)	
3, n(%)	360 (29.5%)	320 (31.5%)	40 (19.6%)	
4, n(%)	601 (49.2%)	449 (44.1%)	152 (74.5%)	
OPS				
0, n(%)	24 (2.0%)	22 (2.2%)	2 (1.0%)	<0.001 (χ^2^ = 96.207)
1, n(%)	177 (14.5%)	173 (17.0%)	4 (2.0%)	
2, n(%)	448 (36.7%)	408 (40.1%)	40 (19.6%)	
3, n(%)	572 (46.8%)	414 (40.7%)	158 (77.5%)	

ARDS, acute respiratory distress syndrome; BMI, body mass index; SOFA, Sequential Organ Failure Assessment score; PNI, prognostic nutritional index; NPS, naples prognostic score; OPS, Osaka prognostic score; IQR, interquartile range.

### 3.3 Comparison of the clinical features between patients with and without ARDS in sepsis patients with diabetes mellitus

Among 868 patients with diabetes mellitus, 754 had no ARDS while 114 were diagnosed with ARDS. The rate of invasive mechanical ventilation utilization was significantly higher in the ARDS group compared with the non-ARDS group (92.1% vs. 32.1%, *p* < 0.001). Patients with ARDS exhibited a statistically lower PNI level than those without ARDS (32.78 (28.08, 37.71) vs. 35.15 (31.25, 40.70), *p* < 0.001). No significant differences were observed in the distributions of the NPS (*p* = 0.732) and OPS (*p* = 0.165) between the two groups (**Table 3**).

**Table 3:** Comparison of the clinical features between patients with and without ARDS in patients with diabetes mellitus.

Clinical characteristics	Total (n=868)	Without ARDS (n=754)	With ARDS (n=114)	*p* (χ^2^/Z)
Gender				
Male, n(%)	645 (74.3%)	558 (74.0%)	87 (76.3%)	0.647 (χ^2^ = 0.277)
Female, n(%)	223 (25.7%)	196 (26.0%)	27 (23.7%)	
Age (years old)				
<60, n(%)	265 (30.5%)	228 (30.2%)	37 (32.5%)	0.663 (χ^2^ = 0.230)
≥60, n(%)	603 (69.5%)	526 (69.8%)	77 (67.5%)	
BMI (kg/m^2^)				
Underweight, n (%)	79 (9.1%)	76 (10.1%)	3 (2.6%)	0.105 (χ^2^ = 4.490)
Normal weight, n (%)	436 (50.2%)	385 (51.1%)	51 (44.7%)	
Overweight, n (%)	312 (35.9%)	280 (37.1%)	32 (28.1%)	
History of smoking				
No, n(%)	708 (81.6%)	614 (81.4%)	94 (82.5%)	0.799 (χ^2^ = 0.069)
Yes, n(%)	160 (18.4%)	140 (18.6%)	20 (17.5%)	
History of alcohol drinking				
No, n(%)	785 (90.4%)	677 (89.8%)	108 (94.7%)	0.122 (χ^2^ = 2.805)
Yes, n(%)	83 (9.6%)	77 (10.2%)	6 (5.3%)	
Hypertension				
No, n(%)	365 (42.1%)	310 (41.1%)	55 (48.2%)	0.155 (χ^2^ = 2.067)
Yes, n(%)	503 (57.9%)	444 (58.9%)	59 (51.8%)	
Invasive mechanical ventilation				
No, n (%)	521 (60.0%)	512 (67.9%)	9 (7.9%)	<0.001 (χ^2^ = 148.618)
Yes, n (%)	347 (40.0%)	242 (32.1%)	105 (92.1%)	
SOFA score	5.0 (3.0, 8.0)	4.0 (3.0, 7.0)	8.0 (5.3, 11.0)	<0.001 (Z=-4.316)
PNI, median (IQR)	34.83 (30.80, 40.13)	35.15 (31.25, 40.70)	32.78 (28.08, 37.71)	<0.001 (Z=-3.920)
NPS				
0, n(%)	4 (0.5%)	4 (0.5%)	0 (0)	0.732 (χ^2^ = 1.949)
1, n(%)	15 (1.7%)	14 (1.9%)	1 (0.9%)	
2, n(%)	69 (7.9%)	61 (8.1%)	8 (7.0%)	
3, n(%)	217 (25.0%)	191 (25.3%)	26 (22.8%)	
4, n(%)	563 (64.9%)	484 (64.2%)	79 (69.3%)	
OPS				
0, n(%)	8 (0.9%)	7 (0.9%)	1 (0.9%)	0.165 (χ^2^ = 4.986)
1, n(%)	72 (8.3%)	68 (9.0%)	4 (3.5%)	
2, n(%)	219 (25.2%)	193 (25.6%)	26 (22.8%)	
3, n(%)	569 (65.6%)	486 (64.5%)	83 (72.8%)	

ARDS, acute respiratory distress syndrome; BMI, body mass index; SOFA, Sequential Organ Failure Assessment score; PNI, prognostic nutritional index; NPS, naples prognostic score; OPS, Osaka prognostic score; IQR, interquartile range.

### 3.4 Logistic regression analysis of the prognostic scores on ARDS in patients with sepsis among patients with and without diabetes mellitus, respectively

**Figure 1:**
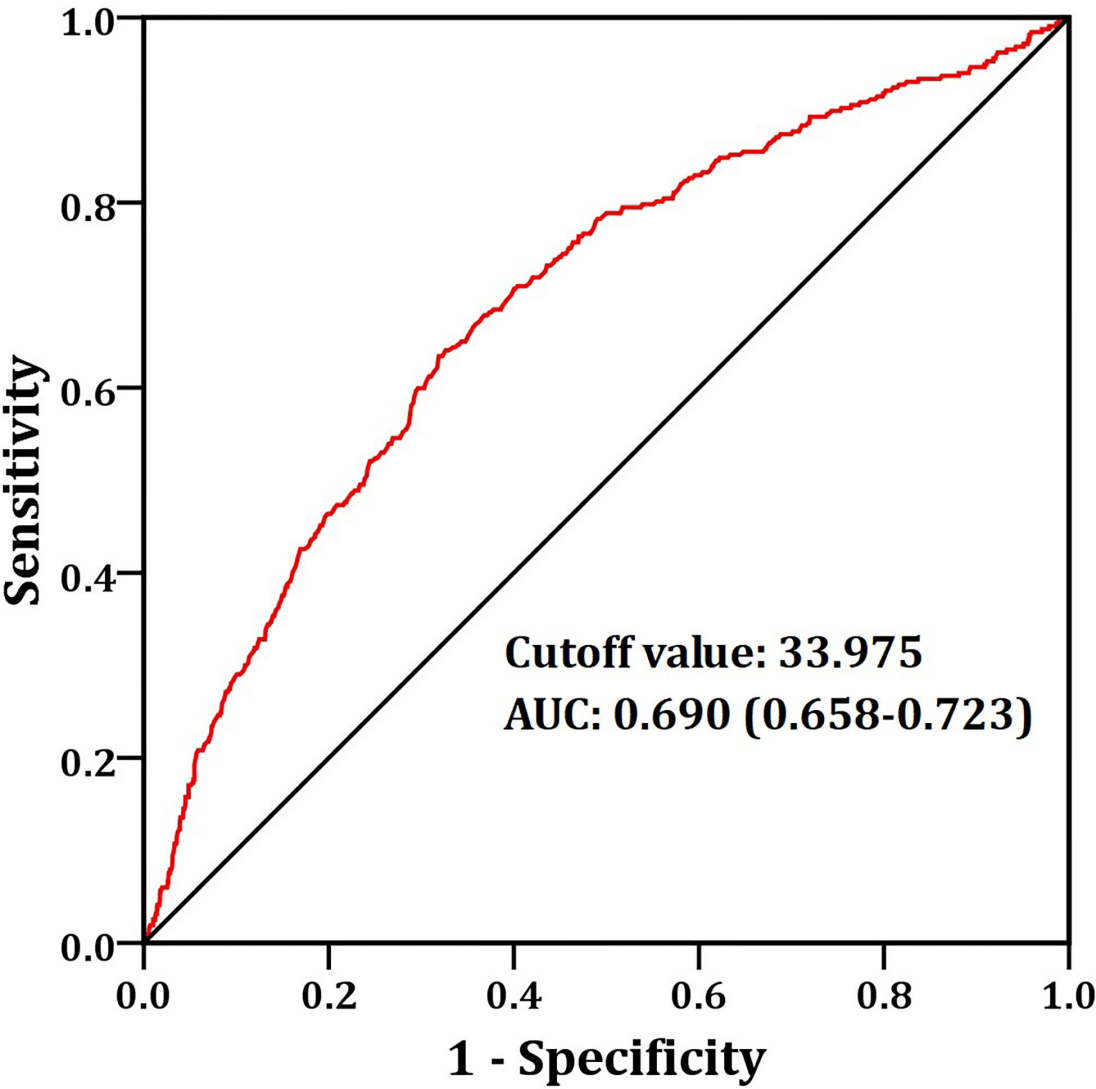
The ROC curve analysis of PNI to distinguish ARDS. ARDS, acute respiratory distress syndrome; PNI, prognostic nutritional index.

When ARDS was set as the endpoint for PNI analysis, receiver operating characteristic (ROC) curve analysis demonstrated that the optimal cutoff value of PNI was 33.975. This threshold yielded a sensitivity of 63.4%, a specificity of 68.1%, and an area under the ROC curve (AUC) of 0.691 (**Figure 1**).

In non-diabetes mellitus patients, univariate analysis results demonstrated that a low PNI (<33.975 versus ≥33.975; odds ratio [OR]: 5.534, 95% confidence interval [CI]: 4.013-7.631, *p* < 0.001), NPS score of 3-4 (versus 0-2; OR: 5.126, 95% CI: 2.812-9.346, *p* < 0.001), and OPS score of 2-3 (versus 0-1; OR: 7.779, 95% CI: 3.402-17.789, *p* < 0.001) were significantly correlated with ARDS. After adjusting for confounding factors including age, gender, BMI, smoking history, alcohol consumption history, and hypertension, multivariate logistic regression analysis revealed that low PNI (<33.975 versus ≥33.975; OR: 3.764, 95% CI: 2.549-5.557, *p* < 0.001), NPS score 3-4 (versus 0-2; OR: 2.537, 95% CI: 1.302-4.944, *p* = 0.006), and OPS score 2-3 (versus 0-1; OR: 3.189, 95% CI: 1.326-7.670, *p* = 0.010) remained independently associated with ARDS (**Table 4**).

**Table 4:** Logistic regression analysis of the prognostic indices on ARDS in patients with sepsis among patients with and without diabetes mellitus, respectively.

Prognostic scoring indices	Non-diabetes mellitus				Diabetes mellitus			
	Crude β/OR (95% CI)	*P* values	Adjusted β/OR (95% CI)	*P* values	Crude β/OR (95% CI)	*P* values	Adjusted β/OR (95% CI)	*P* values
PNI (<33.975 vs. ≥33.975)	5.450 (3.957-7.506)	<0.001	3.720 (2.522-5.487)	<0.001	2.208 (1.476-3.303)	<0.001	2.037 (1.256-3.306)	0.004
NPS (score 3–4 vs. score 0-2)	5.160 (2.830-9.407)	<0.001	2.586 (1.328-5.036)	0.005	1.365 (0.665-2.804)	0.396	0.973 (0.398-2.382)	0.953
OPS (score 2–3 vs. score 0-1)	7.828 (3.424-17.900)	<0.001	3.269 (1.360-7.854)	0.008	2.408 (0.952-6.088)	0.063	1.745 (0.566-5.376)	0.332

ARDS, acute respiratory distress syndrome; PNI, prognostic nutritional index; NPS, naples prognostic score; OPS, Osaka prognostic score; OR, odds ratio; CI, confidence interval.

Adjust for: age, gender, BMI, history of smoking, history of alcohol drinking, and hypertension.

In patients with diabetes mellitus, multivariate logistic regression analysis showed that low PNI (<33.975 vs. ≥33.975, OR: 2.037, 95% CI: 1.256-3.306, *p* = 0.004) was independently associated with ARDS, however, neither NPS nor OPS yielded statistically significant results (**Table 4**).

## 4 Discussion

This study focuses on three convenient prognostic assessment indices based on peripheral blood parameters, namely the PNI, NPS, and OPS, and systematically explored their clinical value in assessing the risk of ARDS in patients with sepsis. The results showed that PNI, NPS, and OPS can effectively predict the risk of ARDS in sepsis patients without diabetes mellitus; however, neither of NPS and OPS showed significant predictive efficacy in sepsis patients complicated with diabetes mellitus. This result reveals the critical modifying effect of diabetic status on predicting the risk of ARDS in sepsis patients, providing important evidence for the development of clinical stratified prediction strategies.

The predictive value of PNI, NPS, and OPS in non-diabetes mellitus patients with sepsis essentially reflects the close correlation between nutritional status and the pathophysiological progression of sepsis. During the infectious state, the hypermetabolic consumption (30) and gastrointestinal dysfunction (31) lead to insufficient nutrient intake, which directly reduces albumin synthesis. Albumin is a key substance for maintaining plasma colloid osmotic pressure and a carrier of inflammatory mediators, hypoalbuminemia exacerbates pulmonary capillary leakage, thereby laying the pathological foundation for the development of ARDS (32, 33). Cholesterol, as a core indicator of lipid metabolism, shows decreased levels that reflect energy exhaustion and deteriorated nutritional status in patients with sepsis (34). Malnutrition, in turn, serves as a crucial predisposing factor for impaired immune function and increased susceptibility to organ damage in these patients (35).

Meanwhile, as a core component of cellular immunity, reduced lymphocyte count indicates impaired anti-infective immune responses (36) and an increased risk of uncontrolled cytokine storm (37), which is a key inducer of the disruption of the alveolar epithelial-capillary endothelial double barrier in patients with ARDS. Abnormal activation of monocytes exacerbates inflammatory infiltration in lung tissue by releasing proinflammatory cytokines, ultimately inducing ARDS (38, 39). Sustained elevation of the inflammatory response directly impairs the pulmonary barrier through mechanisms such as activating neutrophils, releasing neutrophil elastase, and generating reactive oxygen species (ROS) (40–42).

The research conducted by Huang et al. indicated that a low PNI level was associated with an increased risk of neonatal respiratory distress syndrome in premature infants (43). PNI can also serve as a predictor of the mortality risk for patients with ARDS (44). Furthermore, some studies have explored the clinical value of NPS in respiratory system diseases. Wu et al. revealed that people with a high NPS score were at an increased risk of suffering from lung diseases (such as asthma, chronic bronchitis, and obstructive and restrictive lung dysfunction) (45). Zhu et al. found that asthma patients with NPS 3 or 4 scores had a significantly increased risk of all-cause mortality, the NPS has predictive value for all-cause mortality in adults with asthma (29). Some studies found that NPS was a prognostic indicator in patients with acute pulmonary embolism (APE) (46, 47). In addition, although there have been some studies on the role of OPS in the prognosis assessment of cardiovascular and cerebrovascular diseases (16, 48) as well as some cancers (21, 49), its clinical value in respiratory system diseases remains unknown.

Furthermore, another important finding of this study is that neither the NPS nor the OPS exhibited significant predictive value for ARDS in diabetes mellitus patients, which is primarily attributed to the interference of inherent metabolic disorders, immune remodeling, and inflammatory heterogeneity in diabetes mellitus patients with these indicators. The chronic hyperglycemic state in diabetes mellitus patients induces persistent oxidative stress (50) and the accumulation of advanced glycation end products (AGEs) (51). This chronic pathological condition predisposes to premature vascular endothelial injury (52, 53). Diabetes mellitus patients are often complicated with lipid metabolism abnormalities associated with insulin resistance, characterized by abnormal total cholesterol levels (54, 55). This baseline disturbance renders total cholesterol unable to accurately reflect the nutritional depletion status in sepsis. Diabetes mellitus patients exhibit a persistent chronic inflammatory state, with baseline CRP levels higher than those in non-diabetes mellitus populations (56). When sepsis occurs, the correlation between the magnitude of CRP elevation and the intensity of the inflammatory response is significantly weakened, making it unable to accurately quantify the inflammatory storm induced by infection. In addition, diabetes mellitus patients have defects in immune function, characterized by impaired neutrophil chemotaxis and phagocytic capacity (57). These changes in immune cells are not only affected by sepsis but also superimposed on underlying immune disorders, making them difficult to be used as independent indicators for assessing the risk of ARDS.

In contrast, the PNI retains its predictive value in diabetes mellitus patients, which may be associated with the indicator characteristics of albumin. As an important nutritional marker synthesized by the liver, albumin is influenced by factors such as chronic liver diseases (58) and kidney diseases (59). However, in diabetes mellitus patients without severe chronic complications, albumin levels can still reflect the body’s long-term nutritional reserve status. The hypermetabolic state during the acute phase of sepsis accelerates albumin catabolism. Even in diabetic patients with underlying metabolic abnormalities, the acute decrease in albumin levels can indirectly reflect the severity of the disease and the rate of nutritional depletion, thereby correlating with the risk of ARDS (60, 61). In addition, dysregulation of lymphocytes impairs effective pathogen clearance, predisposing individuals to pulmonary infection or systemic inflammatory response syndrome (SIRS). Notably, infection represents one of the primary etiological factors for ARDS (62). The lymphocyte count reflects systemic immune function and remains relatively robust against the metabolic disturbances (such as diabetes mellitus). In contrast, NPS and OPS include multiple metabolic and inflammatory parameters that may be dysregulated in diabetes, thereby weakening their prognostic performance in this population. These findings suggest that simpler nutritional-immune scores such as PNI may maintain better prognostic stability in patients with diabetes mellitus.

The core advantage of this study lies in its focus on septic patients with and without diabetes mellitus, systematically comparing the differences in the predictive performance of three prognostic scores for ARDS risk, clarifying the score selection strategy under different underlying disease states, and providing direct evidence for clinical individualized risk assessment. However, this study has the following limitations. First, the single-center retrospective design may give rise to selection bias; for instance, baseline characteristics of diabetic sepsis patients in our center, such as blood glucose control levels and complication rates, may differ from those in other institutions, which impairs the extrapolability of the results. Second, potential confounding factors including sepsis severity classification and infection sites were not incorporated into the analysis, and these factors may simultaneously affect nutritional scores and the risk of ARDS development. Third, complete data for calculating nutritional risk scores such as Nutrition risk in the critically ill (NUTRIC score) or Nutritional Risk Screening (NRS 2002) score were not available. Although BMI has limited diagnostic value in critically ill patients and cannot solely define obesity or overweight, it was the only available nutritional-related indicator in our database. Therefore, the nutritional status of included patients was not comprehensively evaluated, which may affect the interpretation of the results. Finally, detailed information regarding diabetes duration and HbA1c levels at admission was not systematically collected for all patients in this study. As chronic metabolic disorders and long−term glycemic control may interfere with the performance of clinical scores such as NPS and OPS, the lack of these data limited our ability to further explore their potential impact on the observed loss of predictive efficacy in the diabetic subgroup. Future studies incorporating baseline glycemic control parameters are warranted to validate and refine the predictive value of these scores in diabetic populations.

Based on the findings of this study, in-depth research can be conducted in the following directions in the future. First, multicenter prospective cohort studies should be carried out to enroll patients with sepsis from hospitals of different levels across various regions, which serves to verify the extrapolability of the conclusions drawn from this study; subgroup analyses can further clarify the impacts of factors such as diabetes duration and blood glucose control status on the predictive efficacy of the scoring systems. Second, a combined predictive model integrating prognostic indices, diabetes-specific indicators and lung injury biomarkers should be established to improve the accuracy of ARDS risk assessment in diabetic patients with sepsis. Third, basic experimental studies are required to elucidate the regulatory mechanisms underlying the effects of AGE accumulation and insulin resistance on the nutrition-inflammation-lung injury axis, thereby providing a molecular biological basis for the optimization of scoring metrics.

## 5 Conclusions

This study focuses on the predictive value of the PNI, NPS, and OPS for the risk of ARDS in patients with sepsis. Stratified analysis was performed based on the key clinical feature of whether patients have comorbid diabetes mellitus. PNI, NPS, and OPS as predictive indicators for the risk of ARDS in sepsis patients without diabetes mellitus; however, NPS and OPS lack corresponding predictive value in diabetes mellitus cohorts. The clinical significance of this study lies in providing population-specific evidence for the selection of assessment tools in the risk stratification of ARDS among patients with sepsis.

## Data Availability

The original contributions presented in the study are included in the article/supplementary material. Further inquiries can be directed to the corresponding author/s.
